# Geospatial Social Determinants of Health Correlate with Disparities in Syphilis and Congenital Syphilis Cases in California

**DOI:** 10.3390/pathogens11050547

**Published:** 2022-05-06

**Authors:** Kelly A. Johnson, Robert E. Snyder, Eric C. Tang, Natalie S. de Guzman, Rosalyn E. Plotzker, Ryan Murphy, Kathleen Jacobson

**Affiliations:** 1 Sexually Transmitted Diseases Control Branch, Division of Communicable Disease Control, Center for Infectious Diseases, California Department of Public Health, Richmond, CA 94804, USA; robert.snyder@cdph.ca.gov (R.E.S.); eric.tang@cdph.ca.gov (E.C.T.); natalie.deguzman@cdph.ca.gov (N.S.d.G.); rosalyn.plotzker@cdph.ca.gov (R.E.P.); ryan.murphy@cdph.ca.gov (R.M.); kathleen.jacobson@cdph.ca.gov (K.J.); 2 Division of Infectious Diseases, Department of Medicine, University of California San Francisco, San Francisco, CA 94143, USA; 3 Department of Epidemiology and Biostatistics, University of California San Francisco, San Francisco, CA 94158, USA

**Keywords:** syphilis, congenital syphilis, social determinants of health, geospatial analysis, public health, sexually transmitted infections

## Abstract

Syphilis and congenital syphilis (CS) are increasing in California (CA). From 2015 through 2019, for example, CA cases of early syphilis among reproductive-age females (15–44) and CS each increased by >200%. Certain populations—including people experiencing homelessness, using drugs, and/or belonging to certain racial/ethnic groups—have been disproportionately impacted. We hypothesized that geospatial social determinants of health (SDH) contribute to such health inequities. To demonstrate this, we geospatially described syphilis in CA using the Healthy Places Index (HPI). The HPI is a composite index that assigns a score to each CA census tract based on eight socioeconomic characteristics associated with health (education, housing, transportation, neighborhood conditions, clean environment, and healthcare access as well as economic and social resources). We divided CA census tracts into four quartiles based on HPI scores (with the lowest quartile having the least healthy socioeconomic and environmental conditions), then used 2013–2020 CA sexually transmitted diseases surveillance data to compare overall syphilis (among adults and adolescents) and CS case counts, incidence rates (per 100,000 population or live births), and incidence rate ratios (IRRs) among these quartiles. From 2013 to 2020, across all stages of syphilis and CS, disease burden was greatest in the lowest HPI quartile and smallest in the highest quartile (8308 cases (representing 33.2% of all incidents) versus 3768 (15.1%) for primary and secondary (P&S) syphilis; 5724 (31.6%) versus 2936 (16.2%) for early non-primary non-secondary (NPNS) syphilis; 11,736 (41.9%) versus 3026 (10.8%) for late/unknown duration syphilis; and 849 (61.9%) versus 57 (4.2%) for CS; all with *p* < 0.001). Using the highest HPI quartile as a reference, the IRRs in the lowest quartile were 17 for CS, 4.5 for late/unknown duration syphilis, 2.6 for P&S syphilis, and 2.3 for early NPNS syphilis. We thus observed a direct relationship between less healthy conditions (per HPI) and syphilis/CS in California, supporting our hypothesis that SDH correlate with disparities in syphilis, especially CS. HPI could inform allocation of resources to: (1) support communities most in need of assistance in preventing syphilis/CS cases and (2) reduce health disparities.

## 1. Introduction

Syphilis and congenital syphilis (CS)—the latter defined as in utero transmission of syphilis from parent to unborn child, preventable with appropriate treatment in pregnancy [1]—are on the rise throughout the United States of America (U.S.A.) and particularly in the large and diverse state of California (CA). Despite approaching elimination twenty years ago, U.S. cases of syphilis (among adults and adolescents) and CS increased by an alarming 74 and 279 percent, respectively, between 2015 and 2019 [2]. Mirroring these national trends, CA cases of early syphilis among (1) all adults and adolescents, (2) reproductive-age females (ages 15–44 years, when in utero syphilis transmission is considered most likely to occur) and (3) CS increased dramatically by 75.8, 201.4 and 200.8 percent, respectively, during the same period [3].

Several sociodemographic characteristics—including (1) living in poverty, (2) experiencing homelessness or unstable housing, (3) being incarcerated, (4) having limited or no access to healthcare, (5) using drugs, and/or (6) belonging to certain gender-identity or racial/ethnic groups (at least in part due to stigma, discrimination, and structural racism)—are associated with syphilis and CS [2,4,5,6]. In CA, more than half of people who delivered an infant with CS in 2018 reported having received delayed or no pre-natal care, half reported methamphetamine use, and one quarter reported incarceration or unstable housing/homelessness [4]. In 2019, among Black Californians, the rates of early syphilis among females (36.2 per 100,000 population) and CS (289.4 per 100,000 live births) were each nearly three times higher than the overall statewide rates among Californians at large, regardless of race/ethnicity or gender [3]. 

We hypothesized that social determinants of health (SDH), encompassing the personal, social, economic, and environmental factors that influence health [7]—and often varying by geographic location [5,8,9] —contribute to these observed health inequities in syphilis/CS. Because SDH are often unmeasured and un/underreported in traditional public health disease investigations, we leveraged an existing metric, the CA Healthy Places Index (HPI), to geospatially describe syphilis/CS in the context of community-level SDH. Our overall goal was to assess whether cases of syphilis/CS correlated with less healthy community-level socioeconomic and environmental conditions, a finding which could have important implications for public health programs seeking to allocate sexually transmitted diseases (STD) funding and resources to areas most in need of support for syphilis prevention and control efforts. 

## 2. Methods

CA HPI is a publicly available composite index [10] that assigns a score (from 0 to 1) to each census tract in the state of CA based on community-level conditions associated with health. Each census tract’s HPI score is derived from 25 indicators in 8 socioeconomic domains (education, housing, transportation, neighborhood conditions, clean environment, and healthcare access as well as economic and social resources). Census tracts with lower HPI scores have fewer opportunities for residents to lead healthy lives. Primary sources of data for the HPI included the 2010 Decennial Census and the American Community Survey (2011–2015), among others [11]. The HPI’s development, specific indicators, and strong association with life expectancy (0.58 coefficient of correlation) have been previously described [11,12]. Census tracts are small, contiguous areas defined by the U.S. Census Bureau, each with approximately 2500–8000 residents, serving as proxies for neighborhoods [13]. Census tracts with fewer than 1500 people, and those with more than 50 percent of the population institutionalized (e.g., residing in nursing homes or correctional facilities) are not assigned HPI scores. Roughly 500,000 people (1.3 percent of the state’s residents) live in the 264 census tracts not assigned HPI scores.

We divided CA census tracts into four quartiles based on their HPI scores (with the lowest quartile having the least opportunity to lead a healthy life). We then used the census tracts of residence among syphilis and CS incidents (e.g., people with syphilis/CS) reported to the California Department of Public Health from 2013 to 2020 to connect each incident with an HPI quartile based on their location of residence. Individuals whose reported address belonged to a provider’s office or a correctional facility—or who resided in a census tract not assigned an HPI score—were excluded from our analysis. Syphilis is a reportable disease, and all incident cases of syphilis and CS must be reported to the California Department of Public Health by both providers and laboratories [14,15,16]. While syphilis has historically been predominant among men who have sex with men (MSM)—who still comprise more than half (57 percent) of CA early syphilis cases reported in 2019 [3]—some of the most dramatic recent increases in syphilis cases have occurred among CA females of reproductive age, as described above. We thus included all reported CA syphilis cases in our analysis (regardless of gender or sexual orientation) so as to not exclude any of the sociodemographic groups most impacted by syphilis in our state.

We aggregated demographic and clinical information (age, sex assigned at birth, race/ethnicity, and stage of syphilis) for each reported syphilis/CS case included in our analysis. Los Angeles and San Francisco counties were excluded from this analysis because—unlike other counties in California—they do not routinely report syphilis/CS cases using the centralized state surveillance database (the California Reportable Disease Information Exchange). Based on guidance from the U.S. Centers for Disease Control and Prevention and in accordance with Federal Office of Management and Budget standards, we also created mutually exclusive race/ethnicity categories, i.e., anyone reporting Hispanic ethnicity was classified as Hispanic regardless of race [17,18]. 

We then compared total syphilis/CS case counts and percentages, incidence rates (per 100,000 population for syphilis and per 100,000 live births for CS), and incidence-rate ratios among the four HPI quartiles. To estimate the syphilis incidence rate, we used 2010 census data to define the total population within each census tract, then aggregated the relevant census tracts to determine the total population within each HPI quartile. Each HPI quartile has roughly the same population (approximately nine million residents). Because the number of live births varies from year to year, we used California vital statistics live birth data from 2018, 2019, and 2020 to estimate CS incidence. These data describe every live birth in California each year. We aggregated the number of live births in each census tract, then took the average of these three most recent years and rounded to the nearest whole number to estimate the average total number of live births, again within the combined census tracts that comprise each HPI quartile (Supplemental Table S1). We used chi-squared tests to compare the distribution of syphilis/CS cases by quartile.

Analysis of public health surveillance data is a routine public health activity. This study was submitted to the Committee for the Protection of Human Subjects at the CA Health and Human Services Agency, where it was considered exempt from review.

## 3. Results 

From 2013 to 2020 in CA, there were 1628 cases of CS, 29,520 cases of primary/secondary (P&S) syphilis, 21,152 cases of early non-primary non-secondary (NPNS) syphilis, and 37,934 cases of syphilis of late/unknown duration reported to the California Department of Public Health (exclusive of Los Angeles and San Francisco counties). Of these, respectively, 1371 (84.2 percent), 24,999 (84.7 percent), 18,136 (85.7 percent), and 27,984 (73.8 percent) were geocoded (i.e., identified as a resident of a census tract assigned an HPI score). The median (25/75 percent interquartile range) ages among geocoded cases of P&S syphilis, early NPNS syphilis, and syphilis of late/unknown duration were, respectively, 32 (25–43), 34 (27–46), and 33 (26–43) years (Table 1). 

While the 1371 CS cases included in the analysis were distributed evenly by gender (50.7 percent male and 48.6 percent female), syphilis of all other stages was more common among males (representing 82.2 percent of P&S cases, 80.1 percent of early NPNS cases, and 64.4 percent of cases of late/unknown duration). Hispanic Californians experienced the largest number of syphilis/CS cases in CA (48.2, 37.2, 41.5, and 44.1 percent of cases of CS, P&S syphilis, early NPNS syphilis, and syphilis of late/unknown duration, respectively). Black Californians were disproportionately impacted by syphilis, representing 12.4, 10.4, 8.9, and 10.3 percent each of cases of CS, P&S syphilis, early NPNS syphilis, and syphilis of late/unknown duration (and only comprising six percent of California’s population in 2019 [19]). 

**Figure 1:**
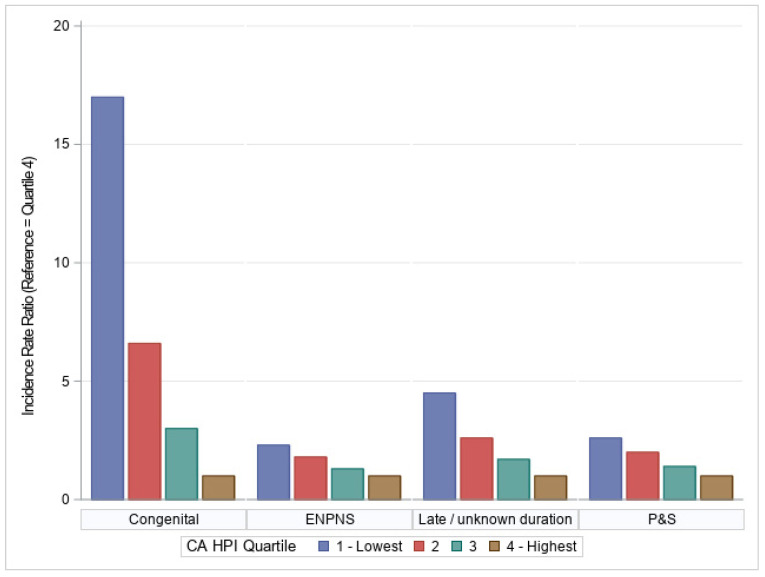
Incidence rate ratios of congenital syphilis, early non-primary non-secondary (ENPNS), late/unknown duration syphilis, and primary and secondary (P&S) syphilis cases reported to the California Department of Public Health, stratified by California Healthy Places Index Quartile, 2013–2020.

Across all stages of syphilis and CS, disease burden was the smallest in the highest HPI quartile, then increased progressively in the lowest HPI quartiles (Table 2, Figure 1). Among CS cases, for example, only 57 cases (4.2 percent of the 1371 total geocoded CS cases) occurred in residents of census tracts that fell into the highest HPI quartile, while 849 cases (61.9 percent) occurred in the lowest quartile. This pattern repeated itself for P&S syphilis (with 3768 cases (15.1 percent) occurring in the highest quartile versus 8308 cases (33.2 percent) in the lowest quartile), early NPNS syphilis (2936 cases (16.2 percent) versus 5724 cases (31.6 percent)), and syphilis of late/unknown duration (3026 cases (10.8 percent) versus 11,736 cases (41.9 percent); all with *p* < 0.001). 

The syphilis/CS incidence rates also increased progressively with decreasing socioeconomic advantage as measured by HPI. CS incidence (cases per 100,000 live births) was 122.0 in the highest HPI quartile versus 2074.6 in the lowest. Similarly, the incidence rates (per 100,000 population) for P&S syphilis, early NPNS syphilis, and late/unknown duration syphilis were, respectively, 55.6, 43.3, and 44.6 in the highest HPI quartile versus 142.8, 98.4, and 201.7 in the lowest quartile. Translated into incidence rate ratios (IRRs), with the highest HPI quartile used as reference, these findings corresponded with IRRs in the lowest quartile of 17 for CS, 2.6 for P&S, 2.3 for early NPNS syphilis, and 4.5 for late/unknown duration syphilis.

## 4. Discussion 

We observed a direct relationship between lower HPI scores and syphilis/CS in CA. Syphilis (of all stages) and CS case counts/percentages and incidence rates were highest in the CA census tracts that scored in the lowest HPI quartile, and lowest in the census tracts that scored in the highest quartile. At the population level—when compared to the census tracts with the highest HPI scores—the rates of CS, late/unknown-duration syphilis, and early syphilis were, respectively, seventeen, over four, and over two times higher in the lowest-scoring census tracts by HPI. These results support our hypothesis that SDH correlate with disparities in syphilis and especially CS in CA. In demonstrating particularly stark differences in CS cases between the lowest and highest HPI quartiles, our findings also suggest that the inequities engendered by differential SDH compound over time, with younger generations in the least healthy communities significantly more likely to be afflicted by CS (with potential consequences including lifelong disability). When comparing the second lowest to the highest HPI quartile, the CS IRR decreased to 6.6 (versus 17 when comparing the lowest and highest HPI quartiles), further highlighting the extent to which individuals in the lowest HPI quartile are disproportionately burdened by syphilis/CS.

Consistent with our findings, the relationships between sexual health, sexually transmitted infections (STIs), and SDH have been widely reported, primarily using individual risk factors as reported via surveys and/or interviews. These individual-level adverse physical, economic, and social determinants of health include: (1) financial hardship, (2) unstable or temporary housing, (3) lack of access to municipal water services or electricity, (4) limited or no education, (5) incarceration, and (6) witnessing neighborhood violence. Each of these social determinants has been associated with reporting more sexual partners, engaging in condomless sex, and having a higher risk of adolescent pregnancy, HIV, and/or other STIs [20,21,22,23,24]. SDH such as lack of access to healthcare (including pre-natal care), transportation challenges, poverty, low health literacy, and lower educational attainment have also specifically been reported among individuals with syphilis [25,26,27]. 

Due to a history of racial segregation and discrimination, many of the SDH that increase an individual’s risk of STIs, including economic hardship (e.g., lower wages and decreased access to health insurance) and less healthy neighborhood conditions (including higher housing density, higher crime rates, and transportation barriers) are more likely to adversely impact some historically marginalized racial/ethnic groups, compounding the risk of infection in these populations [28,29]. These SDH are also likely to impact sexual networks within these populations, making it more likely that an individual belonging to these racial/ethnic groups will have sex with someone else who has an STI such as syphilis [30]. These racial/ethnic disparities were reflected in CA surveillance data: Black and Hispanic/Latinx Californians were disproportionately impacted by syphilis/CS from 2013 to 2020. In addition to race/ethnicity considerations, people living in close physical proximity to one another are perhaps more likely to have sex with others in the same community due to factors such as convenience, social networks, and similar lived experiences, all of which may compound increasing syphilis rates within the sexual networks in specific communities. For public-health STD programs to optimize the allocation of resources and funding to populations most impacted by syphilis/CS and other STIs, it is crucial to understand not only *what* structural SDH are associated with increased risk but also *in which communities* the burden of these adverse SDH is highest. In recent years, there has been increasing interest in such geospatial analyses of SDH. Prior studies, for example, have found population-level associations between lower neighborhood/regional socioeconomic status and health conditions ranging from ischemic heart disease [31] and asthma [32] to HIV [33], COVID-19 [34], and even all-cause mortality [13], among others. 

Fewer studies have geospatially analyzed syphilis/CS morbidity, and none (to our knowledge) have previously used the variety of SDH indicators that make up the CA HPI. Researchers in the Brazilian state of Mato Grosso used six metrics to estimate SDH and describe a municipality-level correlation between the incidence of CS and structural challenges such as poor sanitary conditions and less than eight years of schooling [9]. In the U.S.A., state-level analyses have shown that states with higher percentages of impoverished residents have higher rates of P&S syphilis among MSM [35]. Nationwide analyses have used modeling to predict the U.S. counties most vulnerable to CS [36] and to the emergence of primary/secondary syphilis among women of reproductive age [37], while national CS hospitalization rates have been shown to be independently associated with geographic region in addition to other SDH [38]. Finally, in Chicago, researchers developed a composite scoring system known as the Sexual and Reproductive Health Burden Index, which used eight indicators of sexual and reproductive health (including the incidence of syphilis and other STIs, teen births, infant mortality, and low infant birthweight) to describe which communities in Chicago were most impacted by adverse sexual and reproductive health outcomes [20]. This approach, while potentially useful for STD resource allocation, is more focused on the sexual health outcomes themselves, as opposed to the SDH that drive inequities between communities.

Our analysis adds to this conversation by establishing that CA HPI, with strengths including the incorporation of over twenty SDH indicators and its strong correlation with life expectancy [11], can be used to identify the CA census tracts most impacted both by less healthy socioeconomic/environmental conditions and increased syphilis/CS morbidity. This suggests that STD program interventions—potentially including grant funding, STI clinic creation, mobile van outreach, and/or educational interventions, among others—might best be targeted toward the CA census tracts falling into the lowest HPI quartile. It also implies that a tiered approach, with resources directed to each census tract in proportion to its HPI score, could be a strategy to improve syphilis prevention and control while reducing health disparities in CA and beyond. STI programs could also consider reporting HPI scores—in addition to characteristics such as age, sex, and gender—when describing syphilis/CS epidemiology in public health surveillance reporting (i.e., in annual STD program surveillance and other documents), to more fully describe the epidemiologic landscape. Even outside of STI programmatic interventions, efforts to address underlying disparities that correlate with increased syphilis/CS case counts (and other health conditions) may also be warranted. 

We do not know why the observed IRRs for late/unknown duration syphilis were higher than those for P&S syphilis in the lowest HPI quartile, but we hypothesize that this could be attributable to people in these communities having less access to healthcare and syphilis testing, even when experiencing symptoms (as with P&S syphilis). It is also possible that some individuals in more advantaged HPI quartiles were less likely to be tested for STIs (since providers may be biased in considering these individuals less at risk for STIs), falsely lowering the overall positivity rates in this quartile, albeit this would be unlikely to account for the significant differences between quartiles observed in our analysis.

Our study has several limitations. First, the HPI is based on data curated primarily from 2010 to 2015, while we leveraged STD surveillance data from a different period (2013–2020) to describe the recent burden of syphilis/CS more accurately. It is possible that the socioeconomic status of some CA census tracts has changed during this time in ways that were not captured by our analysis. Additionally, we did not assess whether race/ethnicity were independent predictors of syphilis/CS, but the distribution of race/ethnicity among all stages of syphilis/CS was similar (Table 1). Because we included all syphilis cases (regardless of gender, sexual orientation, or race/ethnicity) in our analysis, we were unable to describe any geospatial differences in syphilis cases among certain sub-populations (such as MSM, females of reproductive age, or people of certain racial/ethnic backgrounds), though this remains an area of interest for future study. 

Our analysis was also limited by missing data. A certain number of syphilis/CS cases were reported without an associated address and were thus unable to be geocoded to a CA census tract with an HPI score. This issue was especially prominent among syphilis cases of late/unknown duration—where approximately 25 percent of cases could not be geocoded—likely due, at least in part, to the public health surveillance process. Once a syphilis case is identified, a disease investigation specialist is assigned to contact the patient and identify recent sexual partners for syphilis testing and treatment. If an investigator is unable to contact a patient to determine symptomology (assuming laboratory test results do not confirm that their syphilis infection was incident within the past year), the patient will automatically be classified as a case of late/unknown duration syphilis. These patients may thus be both: (1) misclassified as to the stage of their syphilis infection (since any symptoms of P&S syphilis would not be reported) and (2) less likely to have an accurately reported address, contributing to their inability to be geocoded. Individuals with syphilis who experience homelessness are also likely underrepresented in our analysis if they were unable to be contacted by a disease investigation specialist and/or did not report a permanent address associated with a specific CA census tract. From 2013 to 2020, CS case reporting in CA was similarly affected by missing data: 10–15 percent of CS cases annually were reported without an address—potentially due to factors including unstable housing reported among their birthing parents and/or the children having been placed into foster care. Future studies could refine this sensitivity analysis using tools to impute infants’ most likely residential HPI quartile, paired with ongoing quality-assurance efforts to ascertain and accurately reflect where infants with CS live, and to better characterize individual maternal sociodemographic characteristics associated with syphilis/CS. 

Finally, because HPI uses aggregate census tract level data as the unit of observation, our analysis is subject to the limitations of all ecological studies. There were likely individuals within each census tract (and on the border of each HPI quartile) whose socioeconomic status and SDH differed from most of the population in their community (or HPI quartile). However, taken as a whole, our analysis establishes a strong community-level correlation between less healthy community conditions as measured by HPI and syphilis/CS. Taking this association even further, when examining CS cases by HPI quartile over time (Supplemental Figure S1), there is evidence that disparities in the lowest two HPI quartiles relative to the others may in fact have widened in recent years. Future analyses could perform more in-depth analysis of syphilis/CS morbidity by HPI quartile over time and/or could further leverage HPI to: (1) evaluate syphilis treatment completion, (2) explore STI testing volumes, and (3) geospatially describe other STIs (including less common manifestations such as neuro/ocular/otic syphilis and/or disseminated gonococcal infections). 

## 5. Conclusions

We identified a strong association between CS/syphilis morbidity and lower HPI scores in California. This finding supports our hypothesis that SDH are correlated with disparities in syphilis and especially CS. Tools such as HPI that allow for geospatial analysis at the community scale could inform allocation of resources and funding to: (1) support communities most in need of assistance with prevention and control of syphilis/CS and (2) reduce health disparities.

## Figures and Tables

**Table 1:** Demographic characteristics among geocoded syphilis cases reported to the California Department of Public Health who lived in a census tract with a California Healthy Places Index Score, * 2013–2020.

	Stage of Syphilis			
	Congenital	Primary/Secondary	Early Non-Primary Non-Secondary	Late, Unknown Duration
Age (median, 25%, 75%)	0	32 (25, 43)	34 (27, 46)	33 (26, 43)
Sex Assigned at Birth				
Male	695 (50.7%)	20,544 (82.2%)	14,667 (80.1%)	18,014 (64.4%)
Female	666 (48.6%)	4273 (17.1%)	3230 (17.8%)	9669 (34.6%)
Other/unknown	10 (0.7%)	182 (0.7%)	239 (1.3%)	301 (1.1%)
Race/Ethnicity				
Hispanic	360 (26.3%)	8398 (33.6%)	5838 (32.2%)	6385 (22.8%)
White	661 (48.2%)	9303 (37.2%)	7522 (41.5%)	12,343 (44.1%)
Black	170 (12.4%)	2589 (10.4%)	1609 (8.9%)	2871 (10.3%)
Asian	32 (2.3%)	1401 (5.6%)	932 (5.1%)	1276 (4.6%)
American Indian/Alaska Native	8 (0.6%)	126 (0.5%)	63 (0.4%)	115 (0.4%)
Other	48 (3.5%)	870 (3.5%)	591 (3.3%)	1010 (3.6%)
Unknown	92 (6.7%)	2312 (9.3%)	1581 (8.8%)	3984 (14.2%)

* All census tracts in California are assigned HPI scores unless there are <1500 residents, or more than 50 percent of the population resides in a congregate facility (e.g., correctional institution, healthcare facility, etc.).

**Table 2:** Number, rate, and incidence rate ratios of geocoded syphilis cases, by California Health Places Index (HPI) quartile reported to California Department of Public Health, 2013–2020.

		Lowest-Scoring Quartile			Highest-Scoring Quartile
HPI Quartile		1	2	3	4
**Congenital Syphilis**	**Cases**	849 (61.9%)	303 (22.1%)	155 (11.3%)	57 (4.2%)
	**Rate/100k Live Births**	2074.6	810.8	361.6	122
	**Incidence Rate Ratio**	17	6.6	3	REF
**Primary Syphilis**	**Cases**	8308 (33.2%)	7247 (28.9%)	5456 (21.8%)	3768 (15.1%)
	**Rate/100k Live Births**	142.8	109.6	77.6	55.6
	**Incidence Rate Ratio**	2.6	2	1.4	REF
**Early Non-Primary Non-Secondary Syphilis**	**Cases**	5724 (31.6%)	5279 (29.1%)	4057 (22.4%)	2936 (16.2%)
	**Rate/100k Live Births**	98.4	79.8	57.7	43.3
	**Incidence Rate Ratio**	2.3	1.8	1.3	REF
**Late/Unknown Duration Syphilis**	**Cases**	11,736 (41.9%)	7687 (27.5%)	5314 (18.9%)	3026 (10.8%)
	**Rate/100k Live Births**	201.7	116.2	75.5	44.6
	**Incidence Rate Ratio**	4.5	2.6	1.7	REF

## Data Availability

The data supporting this analysis are publicly available. CA HPI metrics are published online at https://map.healthyplacesindex.org/?redirect=false (accessed on 28 April 2022). De-identified CA human surveillance data are similarly available via the California Health and Human Services Open Data Portal: https://data.chhs.ca.gov/ (accessed on 28 April 2022).

## Supplementary Materials

The following supporting information can be downloaded at: https://www.mdpi.com/article/10.3390/pathogens11050547/s1. Table S1: Average number of live births by California Health Places Index (HPI) quartile in California*, 2018–2020, Figure S1: Congenital syphilis cases reported to the California Department of Public Health and geocoded to an HPI quartile, by year and HPI quartile, 2013–2020.
