# Supplementary material for: Geospatial Social Determinants of Health Correlate with Disparities in Syphilis and Congenital Syphilis Cases in California
Source: Pathogens. 2022 May 6;11(5):547. doi: 10.3390/pathogens11050547 (PMC9146036; doi:10.3390/pathogens11050547)

Table S1. Average number of live births by California Health Places Index (HPI) quartile in California*, 2018-2020.

HPI Quartile	Live Births
1 Lowest-scoring	40923
2	37369
3	42863
4 Highest-scoring	46717

* Excluding Los Angeles and San Francisco Counties.

Figure S1. Congenital syphilis cases reported to the California Department of Public Health and geocoded to an HPI quartile, by year and HPI quartile, 2013-2020.

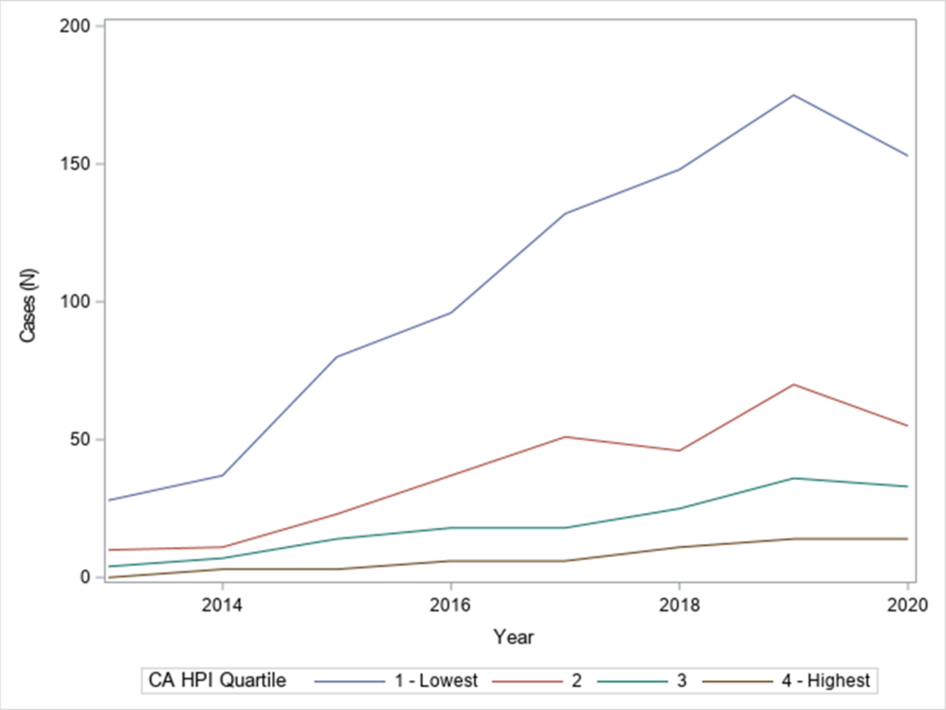

Supplement: Supplementary file 1 [file pathogens-11-00547-s001.zip › pathogens-1685266-supplementary.pdf]
